# The London Provident Dispensaries Council

**Published:** 1907-10-26

**Authors:** H. A. Harben

**Affiliations:** Chairman, London Provident Dispensaries Council.


### THE LONDON PROVIDENT DISPENSARIES COUNCIL.

*To the Editor of* The Hospital.

Sir,—Public attention has been frequently drawn of late years to the overcrowded state of the out-patient departments of our hospitals. Not only does this overcrowding put a great strain upon the resources of the hospitals and cause delay in the treatment of patients, but, what is of far more importance, it implies that many persons who are well able to provide for themselves seek free medical relief at the hospitals for comparatively trifling ailments.

The only means of effectually combating this evil lies in co-operation between the hospitals and provident dispensaries. A provident dispensary places efficient medical attendance within the reach of those wage-earners who, though they may be unable to pay ordinary medical fees, are yet not poor enough to justify them in seeking free medical treatment. A provident dispensary properly so-called should have a membership on the basis of regular payments on insurance principles, a reasonable wage-limit being fixed as a condition of membership, and its medical staff should be sufficient in number to preclude the suggestion that it is established for the benefit of one or two practitioners. It should, moreover, be managed by a committee, upon which the medical staff should be represented, and a properly audited balance sheet should be published annually.

While there exist in various parts of London many excellent dispensaries established upon the principles I have indicated, they are not sufficiently numerous or well-distributed to be able to undertake the treatment of the respectable and provident poor of the whole of the Metropolis. In these circumstances the London Provident Dispensaries' Council has been lately formed as the outcome of a conference of the provident dispensaries of London. Its objects are to circulate information on the provident medical movement among the medical profession and the public at large, and to promote the establishment over the whole area of London of well-equipped and well-regulated provident dispensaries. It will endeavour, while promoting the independence and individuality of existing provident dispensaries, to promote co-operation between them, and to afford opportunities for comparing their methods of administration. It will also endeavour to promote that co-operation between provident dispensaries and hospitals which is essential for the relief of the out-patient departments of the latter institutions. Every provident dispensary of London has been asked to send a representative—medical or lay—to the Council, and it is intended to add a few co-opted members, including nominees of the British Medical Association.

It is hoped that all those who are interested in existing dispensaries and all who desire to encourage habits of thrift and self-reliance among the poor will endeavour to further the objects of the London Provident Dispensaries Council.

Further information will be gladly supplied by the Hon. Secretary, Charles H. Warren, and may be obtained at the temporary offices of the Council at 5 Lamb's Conduit Street, W.C.

Yours, etc.,
H. A. Harben,
Chairman, London Provident Dispensaries Council.

